# Ricin Toxicokinetics and Its Sensitive Detection in Mouse Sera or Feces Using Immuno-PCR

**DOI:** 10.1371/journal.pone.0012858

**Published:** 2010-09-22

**Authors:** Xiaohua He, Stephanie McMahon, Thomas D. Henderson, Stephen M. Griffey, Luisa W. Cheng

**Affiliations:** 1 Foodborne Contaminants Research Unit, Western Regional Research Center, United States Department of Agriculture, Agricultural Research Service, Albany, California, United States of America; 2 Comparative Pathology Lab, School of Veterinary Medicine, University of California Davis, Davis, California, United States of America; New Mexico State University, United States of America

## Abstract

**Background:**

Ricin (also called RCA-II or RCA_60_), one of the most potent toxins and documented bioweapons, is derived from castor beans of *Ricinus communis*. Several *in vitro* methods have been designed for ricin detection in complex food matrices in the event of intentional contamination. Recently, a novel Immuno-PCR (IPCR) assay was developed with a limit of detection of 10 fg/ml in a buffer matrix and about 10-1000-fold greater sensitivity than other methods in various food matrices.

**Methods and Findings:**

In order to devise a better diagnostic test for ricin, the IPCR assay was adapted for the detection of ricin in biological samples collected from mice after intoxication. The limit of detection in both mouse sera and feces was as low as 1 pg/ml. Using the mouse intravenous (iv) model for ricin intoxication, a biphasic half-life of ricin, with a rapid t_1/2_α of 4 min and a slower t_1/2_β of 86 min were observed. The molecular biodistribution time for ricin following oral ingestion was estimated using an antibody neutralization assay. Ricin was detected in the blood stream starting at approximately 6–7 h post- oral intoxication. Whole animal histopathological analysis was performed on mice treated orally or systemically with ricin. Severe lesions were observed in the pancreas, spleen and intestinal mesenteric lymph nodes, but no severe pathology in other major organs was observed.

**Conclusions:**

The determination of *in vivo* toxicokinetics and pathological effects of ricin following systemic and oral intoxication provide a better understanding of the etiology of intoxication and will help in the future design of more effective diagnostic and therapeutic methods.

## Introduction

Ricin is derived from the seeds of the castor plant *Ricinus communis*, and comprises approximately 1% to 5% of the weight of the castor bean mash that remains after oil extraction [1]–[3]. The high toxicity and large quantities of raw materials readily available to produce toxin are a major bio-terror concern [3]–[5]. In its crude form, ricin is often found in association with a lectin, *Ricinus communis* agglutinin (RCA-I or RCA_120_), which has hemagglutinating activity but greatly reduced toxicity compared to ricin. RCA-I is a tetramer containing two non-covalently bonded ricin-like dimers. Ricin is a member of the Type II ribosome inactivating proteins, known as dimeric or AB toxins, of which shiga and shiga-like toxins are members [6]. The two ricin polypeptide chains have molecular weights of 30 kDa (A) and 33 kDa (B), and are normally linked by a single disulfide bond. The ricin A chain is a highly active *N*-glycosidase that initiates depurination and cleavage of 28S ribosomal RNA at position 4324 [7]. The cleaved RNA is no longer capable of binding Elongation Factor 2, which is needed for protein synthesis [8], [9]. Once in the target cell, a single ricin molecule can inactivate more than 1500 ribosomes per minute, ultimately resulting in cell death. The ricin B chain binds to galactose-containing glycoproteins or glycolipids on the surface of target cells and helps facilitate toxin entry into cells via receptor-mediated endocytosis.

The levels of toxicity vary depending on the route of ricin exposure. The mean lethal dose (LD_50_) in mice is approximately 1000–fold lower by injection or inhalation than by oral administration [5]. The lethal dose for an adult human is about 0.35–0.7 mg by inhalation, whereas the lethal oral dose has been estimated to be between 1 to 20 mg of ricin/kg body weight. The large discrepancy in oral and systemic toxicity is likely due to the harsh digestive conditions found in the stomach and epithelium and innate immune barriers present in the intestinal tract [10]. Currently, there is no effective treatment available for ricin intoxication. To develop methods for treatment of ricin intoxication, sensitive, accurate, and robust assay formats that can monitor ricin in human biological samples in a timely manner are required [11]. However, very small amounts of ricin are required to cause lethality in small animals, and most existing methods for the detection of ricin are not robust or sensitive enough for the detection of ricin in biological samples such as sera or animal waste. Thus, little research data are available on the *in vivo* toxicokinetics of ricin and the dynamics of ricin following intoxication. Previous attempts at determining the distribution and fate of toxin employed radiolabelled ricin [12], [13]. While the location and intensity of radioactive label (either from intact or degraded toxin) can be discerned**,** this method is not practical for diagnostic uses in human biological samples.

Because of the high bioterrorism risk concern over ricin, more and more recent research efforts have been directed to the design of sensitive methods for the detection of this toxin in both buffer and complex food matrices. They range from the common ELISA techniques to quantitative PCR, multiplex detection, and even mass spectrometry [11], [14]–[20]. Several of these *in vitro* methods allow the detection of ricin at concentrations as low as 50–100 pg/ml. Recently, we developed a novel IPCR assay for ricin. This assay combines the advantages of the flexible and robust immunoassays with the exponential signal amplification power of PCR [21]. It allows for the detection of as little as 10 fg/mL of ricin in PBS buffer, 10 pg/mL in liquid egg and milk, and 100 pg/mL in ground beef extracts. In this study, we applied the novel IPCR assay to monitor the distribution dynamics of ricin after oral or systemic intoxication, determine the biologic half-life of ricin in sera, and evaluate the potential to use feces or sera as diagnostic samples for ricin intoxication. The data obtained enriched our knowledge of the process of ricin intoxication and the validated IPCR assay provides a new diagnostic tool for the detection of ricin in biological samples.

## Materials and Methods

### Materials

Pure ricin (RCA-II, Cat. # L1090) and polyclonal goat anti-*ricinus communis* agglutinin I and II (Cat. # AS-2084) were purchased from Vector Laboratories (Burlingame, CA) and stored at 4°C. Soluble crude ricin extracts were prepared from castor beans as described previously [14]. Briefly, castor beans, accession PI215769, were obtained from USDA-GRIN, Southern Regional Plant Introduction Station (Griffin, GA). Acetone powder was prepared as described by Tewfik and Stumpf with slight modification [22]. Dry seeds, with shells removed, were ground in liquid nitrogen to a fine powder with mortar and pestle. Cold acetone (5–10 mL/g powder) was then added to form a uniform homogenate. The resulting mixture was filtered by suction on a large Buchner funnel, washed 3 times with cold acetone, and 2 times with small portions of dry ethyl ether. The freshly prepared powder was dried overnight in a vacuum desiccator over P_2_O_5_, and stored at –20°C for future use. Crude ricin powder was solubilized in phosphate-buffered saline (PBS: pH 7.3). The ricin content of crude samples was estimated by SDS-PAGE followed by Coomassie staining and densitometry comparison of unknown RCA-II samples with a RCA-II protein standard curve (data not shown). CD-1 mice (4.5-weeks old, female) were obtained from Charles River Laboratories (Hollister, California).

### Systemic and oral mouse models

Randomly grouped mice (weights of 18–20 g) were inoculated intraperitoneally (ip) or intravenously (iv), via the lateral tail vein, with 100 µl of toxin dosage sample; or orally, via gavage with a popper feeding needle, with 100–200 µL of toxin dosage sample. For the determination of mean lethal dose (LD_50_), groups of 10 mice were used at each dosage level and 4 or 5 dosage levels were tested per experiment. Animals were monitored for at least 9 days for signs of intoxication or death. Mice that were clearly moribund were euthanized and counted as dead. LD_50_'s were calculated using the Weil method [23]. Animal studies conducted herein, adhere to institutional guidelines for husbandry approved by the Animal Care and Use Committee of the U.S. Department of Agriculture, Western Regional Research Center.

### Histopathology of ricin intoxication in mice

Mice were dosed with lethal doses of pure or crude ricin via ip or ig routes (0.006 mg of ricin by ip or 0.75 mg of ricin by ig). Necropsy was performed on ricin treated or non-treated control animals at indicated times (day 1 or 2). Organs (liver, pancreas, kidneys, spleen, lymph nodes, heart, lung, brain, stomach, cecum, small and large intestines, colon, and thymus) were preserved using 10% formalin and processed into paraffin blocks. 5 µm tissue sections were then stained with hematoxylin and eosin. All samples were blinded before histological pathology exams. Tissue processing, embedding, sectioning, staining and analysis were performed at the University of California Davis.

### Serum toxicokinetic measurements

Blood was collected from mice (weights of 19.5–20 g) from the submandibular site into serum collection tubes with gel separators (BD Biosciences) at each time or dosage point. Samples were incubated on ice for at least 30 min before centrifugation at 3000 *g* for 10 min to isolate sera from the cellular fraction. Collected sera were aliquoted and stored at −80°C before analysis. For the mouse systemic toxicokinetic measurements, random groups of 5–10 mice were injected iv via the lateral tail vein with 5 µg/mouse of ricin holotoxin in phosphate saline buffer (PS: 10 mM phosphate buffer, pH 7.8, 150 mM NaCl). In the oral model, 10 mice were treated with 1 mg ricin in 200 µl PS buffer via gavage using round-ended Popper needles. Biological samples such as urine and feces were collected by placing animals in metabolic caging or in bedding-free caging with wire rack bottoms for 30 min prior to the indicated times. Standard curves and unknowns were plotted and determined respectively using a non-linear regression, with variable slope sigmoidal curve fitting model provided by the GraphPad Prism statistics software. Biologic half-life of ricin was determined by calculating two-phase exponential decay over time (2.5 min to 480 min) using the same program.

### ELISA

ELISA was performed as described previously [21]. Briefly, microtiter wells were coated with an anti-ricin mouse monoclonal antibody mAb1642 at 4 µg/mL and incubated overnight at 4°C. After blocking, 100 µL of toxin standards and samples were added to each well and the plates were incubated at room temperature (RT: 23–25°C) for 1 hour. After washing, 100 µL of goat anti-ricin polyclonal antibody at 1 µg/mL was added to each well and the plates were incubated for 1 hour at RT. The plates were then washed with water and 100 µL of 1∶20,000 diluted donkey-anti-goat-HRP conjugate (Promega, Madison, WI) was added to each well. After incubation for 1 hour at RT, the plates were washed and 100 µL of Enhanced K-Blue substrate (Neogen Corp., Lexington, KY) was added to detect bound antibodies. The limit of detection (LOD) is defined as the lowest concentration used for the standard curve at which the average absorbance reading at 450 nm is higher than that of the negative control (NC) plus three fold standard deviation (SD).

### Immuno-PCR

IPCR was performed as described for Sandwich IPCR with minor modifications [21]. Briefly, 30 µL of goat anti-ricin polyclonal antibody (4 µg/mL) was used to coat microtiter wells overnight at 4°C. After blocking non adsorbed sites with 150 µL of blocking buffer for 1 hour at RT, 30 µL of ricin standards or samples were added to each well and the plates were incubated at RT for 1 hour. The plates were washed, 30 µL of streptavidin-conjugated goat anti-ricin polyclonal antibody (80 ng/mL) was added to each well and incubated at 37°C for 30 min. After washing, 30 µL of biotinylated DNA marker (0.5 ng/µL) was added to each well and incubated at RT for 30 min. After thorough washing to remove unbound DNA, the bound DNA was detached from the immuno-complex by restriction enzyme digestion with *BamHI*. A 6 µL digested aliquot was then used as a template in real-time PCR (final 20 µL volume). It should be noted that similar to other immunological assays, the sensitivity and specificity of IPCR depend on the quality of the antibodies. False positive results could be obtained when low specificity antibodies are used.

### PCR Data analysis

The cycle threshold (Ct) used was calculated automatically by the instrument and represents the first PCR cycle at which the fluorescent reporter signal exceeds the signal of a given uniform ‘threshold’ suggested by the instrument software. Duplicate experiments were performed. Mean values and SD of Ct for the intra-assay triplicate measurements of IPCR were calculated using Microsoft Excel software. A linear regression fit of the data was determined, along with 95% confidence limits, using the GraphPad Prism 5.3 software (La Jolla, CA). The Ct value is inversely proportional to the concentration of ricin. The lower the ricin concentration is, the higher the Ct value will be. LOD of the IPCR assay is defined as the lowest concentration used for the standard curve at which its Ct value falls below the average Ct value of the negative control minus 3 SD [24]. It is important to note that the LOD is not absolute, but is calculated relative to the value of the negative control. For the calculation of the LOD in the sera and feces, these matrices were used for their calibration curves. The assay recovery in sample matrices is calculated by dividing the concentration quantified by the nominal concentration spiked into sample and then multiplying this value by 100.

### Sample preparation

Serum calibration curves were prepared by spiking 10 µl of 10-fold serial dilutions of ricin in 90 µl of sera samples; the samples were then diluted at a 1∶9 ratio in PBS to remove any matrix effect before IPCR analysis. Feces standard curves were prepared by spiking 10-fold serial dilutions of ricin in PBS to feces pellets (1 g of feces in 10 mL PBS). The samples were homogenized, and then centrifuged for 5 min at 10,000 *g*. The supernatants were diluted at a 1∶9 ratio in PBS and used for IPCR analysis.

### Polyclonal antibody neutralization of ricin intoxication

Random groups of 5 or more mice were treated iv with 5 µg/mouse of ricin in PS buffer. 100 µg of polyclonal goat anti-ricin antibodies diluted in PBS buffer were introduced iv at 30 min prior to toxin or at indicated time intervals post-intoxication. For neutralization of ricin following oral intoxication, random groups of 10 mice were treated with 1 mg ricin (200 µl of 5 mg/ml toxin stock solution) via gavage. 80 µg of goat anti-RCA I and II was diluted in PBS and injected iv either prior to or after intoxication at indicated times. All mice were monitored for at least 12 days for symptoms of intoxication, weight gain/loss or death. Statistical significance was determined using Log rank tests of survival curves using the GraphPad Prism statistics software where *p*<0.05.

## Results

### *In vivo* toxicity of ricin

The *in vivo* toxicity of pure ricin was determined for both systemic (via ip or iv intoxications) or oral routes of intoxication in adult CD-1 outbred mice (Table 1). Toxicity was expressed as mean lethal dose (LD_50_) values. The lower the units (mg ricin/kg), the higher the toxicity, as less toxin was required for lethality. In this study, the mouse LD_50_ for pure ricin in the oral or intragastric (ig), intraperitoneal (ip) and intravenous (iv) routes of intoxication were 29 mg/kg, 0.023 mg/kg and 0.008 mg/kg respectively.

**Table 1:** LD_50_ of pure ricin in mouse toxicity tests.

Route of administration	LD_50_ (mg/kg) a
Oral	29±5
Intraperitoneal (ip)	0.023±0.002
Intravenous (iv)	0.0075±0.0007

a Ricin holotoxin content ± SEM.

### Histopathology of ricin intoxication

**Figure 1:**
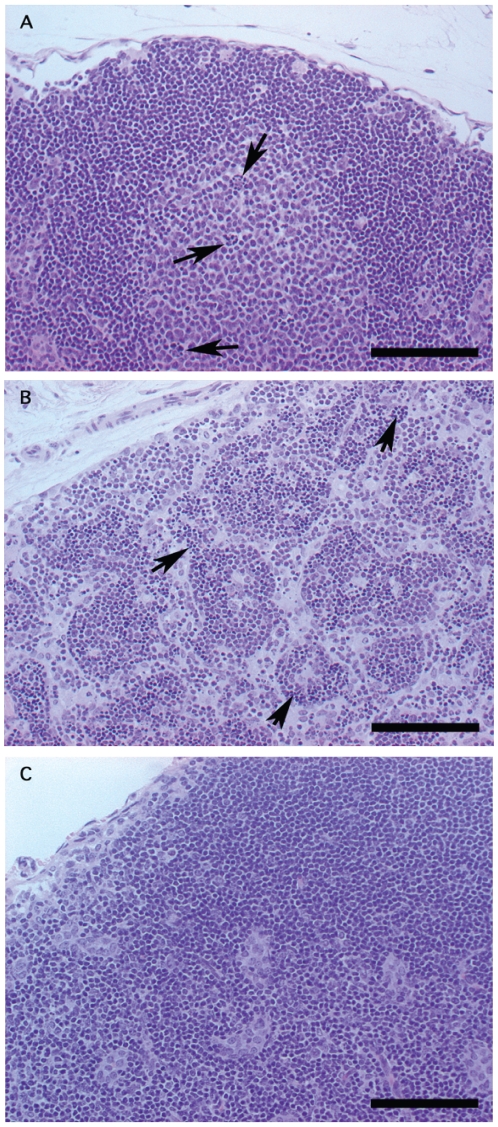
Sections of lymph nodes in mice ip treated with pure ricin were stained with hematoxylin and eosin. A. Lymph node at day one post-intoxication. Several tingible body macrophages (arrows) are present within a follicle consistent with lymphoid reactivity. B. Lymph node at day two post-intoxication. There is moderate lymphoid necrosis characterized by clusters of small, dark basophilic, round apoptotic bodies (arrows) admixed with intact lymphocytes. C. Lymph node from non-treated control mouse for comparison. Bar = 100 µm.

Mice were dosed ip with 0.006 mg, or about 13 LD_50_ units of ricin per mouse. Lethality was observed between days 2–4 after intoxication. Necropsies were performed on day one when animals were ruffled and slow in motion, and on day two on moribund mice. On day one post-intoxication, tissue sections revealed mild lymphoid reactivity as characterized by the presence of tingible body macrophages in the mesenteric lymph nodes (Fig. 1A) and spleen. Mild pancreatic necrosis was also present. On day two post-intoxication, severe to moderate lymphoid necrosis was observed in the thymus, spleen and lymph nodes (Fig 1B). Other organs or tissues had no significant changes compared to untreated mice (Fig. 1C). Moderate epithelial acinar necrosis in the pancreas was also observed. Orally treated mice showed similar lymphoid reactivity and necrosis in the lymph nodes, spleen and thymus as ip treated mice (data not shown). In several mice, gastric necrosis or acute enteritis was also observed.

### IPCR detection of ricin in biological samples

**Figure 2:**
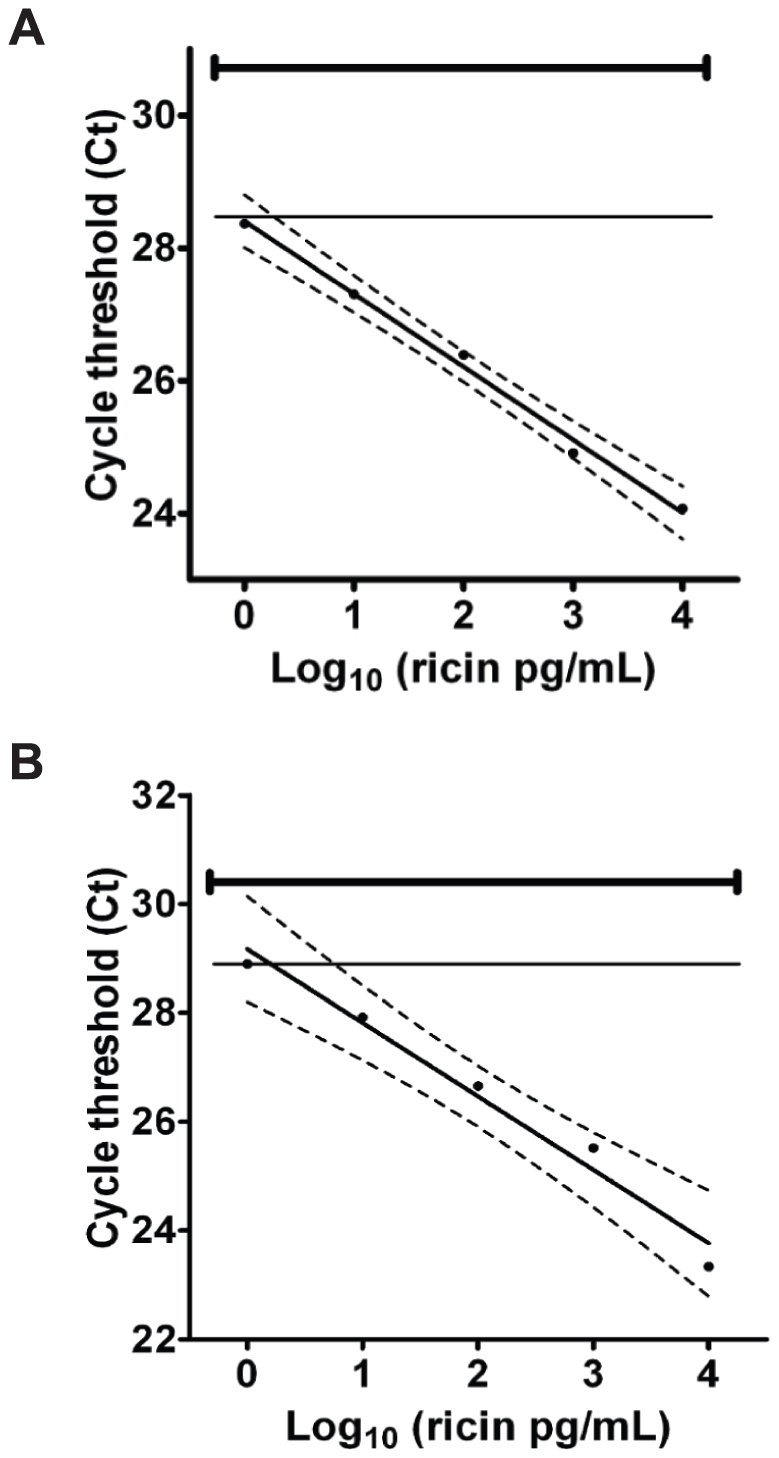
Standard curves for IPCR detection of ricin in sera and feces. Ct values were plotted against log ricin concentrations (pg/mL) for three replicate samples in mouse sera (A) and feces (B). The linear regression of the calibration curve has a correlation coefficient of r^2^ = 0.99. The dashed lines are the upper and lower 95% confidence limits. The solid horizontal thick line denotes the average blank Ct value, with the standard deviation drawn at each end of this line. The solid horizontal thin line intersecting the linear regression line indicates the detection threshold of the IPCR assay as defined in the text.

Our previous results showed that a sandwich IPCR improved ricin detection sensitivity 10- to 1,000-fold in spiked ground beef, milk and chicken eggs when compared to an analogous ELISA assay [21]. In this study, we evaluated the same IPCR protocol on sera and feces samples from mice. Sera and feces were collected from healthy mice. Processed samples were spiked with ricin before a 10-fold serial dilution. Three replicate IPCR measurements were carried out for each serial dilution of ricin (concentration range: 0.1 to 100,000 pg/mL) plus an un-spiked blank or negative control. Figure 2 shows the average cycle threshold (Ct) values obtained by IPCR versus the log of the concentration of ricin for three replicate measurements of ricin in sera (A) and feces (B). The limit of detection (LOD) for sera and feces using this assay is 1 pg/mL based on the linear regression and 95% confidence limits derived from the sample data. The log linear quantification range of the assay is 1 to 10,000 pg/mL. A qualitative detection of ricin in sera lower than 1 pg/mL is also possible due to a clear separation of the signals at 0.1 pg/mL (Ct value ± SD  = 28.50±0.24) from the negative control (Ct ± SD  = 30.79±0.19). Table 2 shows the recovery of ricin from spiked mouse sera and feces samples. At ricin levels ranging from 1–10,000 pg/mL, the recovery from serum was at least 98.2%, and recovery from feces was at least 89.7%.

**Table 2:** Percent (%) recovery of ricin from mouse sera and feces.

Spike level (pg/mL)	1	10	100	1,000	10,000
Serum	98.4±0.9	103.4±2.4	107.1±0.8	109.4±0.9	98.2±0.1
Feces	101.2±0.2	112.4±0.3	99.7±0.8	89.9±0.9	89.7±2.0

Data is the average of three replicates ± one standard deviation.

The detection sensitivities of the IPCR assays for ricin in mouse sera and feces were compared with a standard ELISA method (Table 3). The detection threshold for ricin in mouse sera and feces by sandwich ELISA was about 10 ng/mL; about 4 orders of magnitude less than the 1 pg/mL sensitivity of the IPCR method.

**Table 3:** Detection of ricin in mouse sera and feces using IPCR and sandwich ELISA.

Spike level	IPCR (average Ct ± s.d.)		ELISA (average A450 ± s.d.)	
ng/mL	Serum	Feces	Serum	Feces
0	30.79±0.19	30.02±0.03	0.14±0.01	0.14±0.01
0.001	28.37±0.14	28.90±0.67	n/a	n/a
0.01	27.31±0.09	27.92±0.50	n/a	n/a
0.1	26.39±0.15	26.66±0.56	0.14±0.01	0.14±0.01
1	24.91±0.02	25.52±0.92	0.17±0.01	0.15±0.01
10	24.08±0.04	23.34±1.00	0.47±0.01	0.27±0.01
100	n/a	n/a	1.08±0.01	0.96±0.01
1,000	n/a	n/a	1.12±0.03	1.51±0.04

Data is the average Ct value and absorbance reading at 450 nm of three replicates ± one standard deviation (s.d) from IPCR and ELISA. n/a, not applicable.

In an attempt to develop a diagnostic tool for oral ricin intoxication, mouse feces were collected after oral inoculation of increasing doses of ricin over time (Table 4). The presence of ricin was determined by IPCR. Feces from mice that did not receive ricin treatment were used as negative controls. Samples with average Ct values of three replicate measurements below that of the negative controls minus 3 standard deviations were considered as ricin positive. Ricin can be detected in feces of mice fed at least 10 µg of ricin per mouse, a non-lethal dose. At higher doses (up to1 mg/mouse), the presence of ricin was clearly found in feces deposited within the first 2 hours after intoxication, while at lower doses (400 µg/mouse or less), ricin was detected frequently in feces deposited after 2 hours post intoxication. In addition, ricin was still detectable in feces deposited between 6 and 24 hours post inoculation at doses of 400 µg/mouse and 1 mg/mouse.

**Table 4:** Detection of ricin in mouse feces after oral administration using an IPCR assay.

			Hours after oral administration		
Amount of ricin fed (µg/mouse)	Control	0–2 h	2–4 h	4–6 h	6–24 h
1	0/5	1/5	1/5	1/5	ND
10	0/5	0/5	4/5	4/5	ND
100	0/3	1/3	3/3	3/3	0/3
200	0/3	1/3	3/3	3/3	0/3
400	0/3	1/3	3/3	3/3	3/3
1000	0/7	6/7	6/7	6/7	6/7

Data indicates the numbers of mice determined by IPCR as ricin positive in feces versus the numbers of mice tested. ND, not determined.

### Systemic ricin intoxication

**Figure 3:**
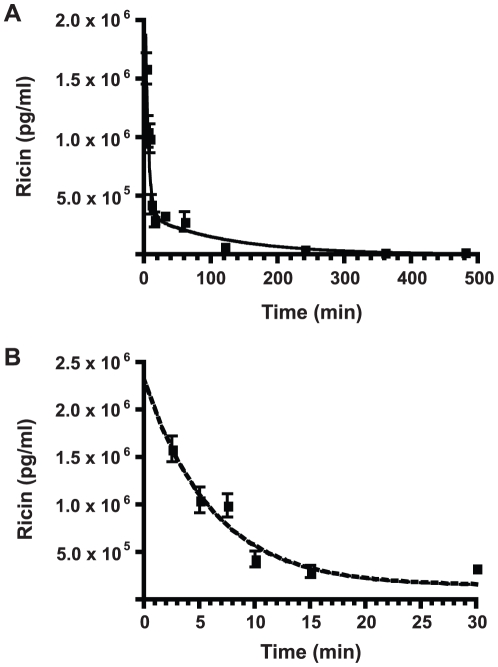
Serum toxicokinetics of ricin. A. Concentrations of ricin in mouse sera over 480 min post -intoxication. Ricin content was determined by IPCR. B. Concentrations of ricin in mouse sera from 2 to 30 min post-intoxication illustrating early and rapid sequestration of ricin with a t_1/2_α half-life of 4 min. Each point in the graph represents the mean value ± SEM. N≥5.

Ricin toxin toxicokinetics data is needed to understand the biological activity of natural ricin. *In vivo* data from mice have been difficult to obtain due to the lack of sensitive methods for the detection of minute quantities of ricin in the complex matrices of biological samples. Our new IPCR method is an amenable system for testing biological samples. To determine the toxicokinetics of ricin following systemic intoxication, we applied this IPCR method to analyze collected sample sets of at least 5 mice challenged iv with a 5 µg/mouse dose or about 33 mouse LD_50_'s of ricin. Sera were collected from each set of mice over time and the concentration of ricin at each time point was determined from a standard curve used in the IPCR. Figure 3A shows a graph of sera concentrations of ricin plotted over time. The first 30 min were re-plotted on a Fig. 3B for a closer look of the early time points. A sharp drop in blood ricin levels was observed within the first 10 min of toxin introduction. This initial phase or alpha half-life (t_1/2_α) was determined to be 4 min (Fig 3B). A slower phase or beta half-life (t_1/2_β), was determined to be approximately 86 min.

Free toxin in sera could potentially be neutralized and cleared by anti-toxin antibodies. In previous work, serum toxicokinetics of the botulinum neurotoxin (BoNT) serotype A were compared in parallel with antibody neutralization of toxin [25]. Decreasing levels of BoNT/A present in sera over time (i.e.: increased absorption of toxin) correlated directly with a decrease in antibody neutralization when anti-BoNT/A antibodies were introduced at subsequently longer times post-intoxication. Thus antibody neutralization assays could help predict the presence of toxins available in sera.

**Figure 4:**
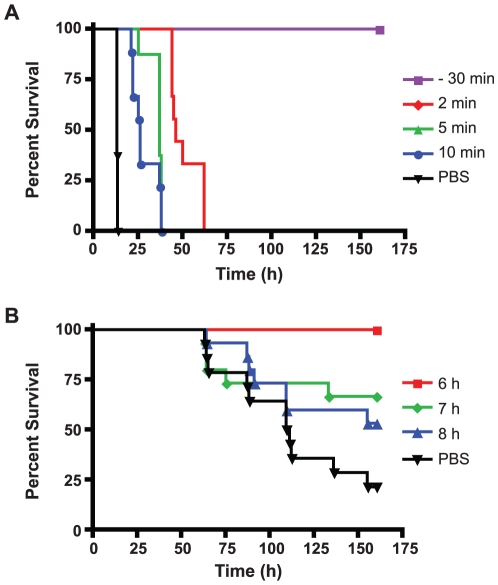
Neutralization of ricin with polyclonal ricin antibodies. Survival percentage of mice dosed iv (A) or ig (B) with ricin were plotted over 160 h post-intoxication. Goat anti-ricin antibodies were introduced iv either prior-to or post-intoxication at indicated times. PBS was injected into mice as no treatment control. Comparison of survival curves with log rank test yielded statistical significance of *p*<0.001 and *p*<0.0144 for A and B respectively.

Using a similar antibody neutralization assay, neutralization of ricin in sera was successfully achieved using a goat polyclonal antibody to ricin. Mice treated iv with 100 µg of antibodies 30 min prior to intoxication with 5 µg of ricin by iv were completely protected from death (Fig. 4A). Addition of the same concentration of antibodies to ricin-treated mice at 2, 5 and 10 min after intoxication conferred some degree of protection as shown by the increase of time-to-death but failed to rescue mice from death. Mice treated at 2 min with antibodies survived the longest with a median survival time of 46 h, followed by median survival times of 37 h and 26 h for mice treated with antibodies at 5 and 10 min respectively. Mice treated with antibodies at 15 min or later (data not shown) showed similar time-to-death curves as the no antibody (PBS only) negative control, which had median survival times of 13 h. A log rank test comparison of survival curves showed statistically significant differences (*p*<0.0001).

### Oral ricin intoxication

The oral intoxication mouse model was used to assess the transit time of ricin from the stomach to the bloodstream. Mice were treated orally with 1 mg or about 1.7 LD_50_ of ricin. The time-to-death for mice treated post-intoxication with control PBS varied from 50 h to 170 h (Fig 4B) with a median survival time of 110 h. Because the transit time varied greatly among animals and the blood clearance rate of ricin can be very rapid as shown in the section above (t_1/2_α of 4 min), it was not possible to obtain consistent data from sampling of mouse sera alone. Far too many mice would be required to obtain enough statistically significant data from close time points. We therefore used an oral antibody neutralization assay to estimate the time it took ricin to traffic from the intestinal tract to the blood stream. Ricin that crossed the intestinal barrier into the bloodstream would be absorbed and distributed quickly, and only when enough neutralizing antibodies against ricin are present prior to arrival of ricin may mouse survival or increased survival time occur.

No deaths occurred when intoxicated mice were treated with 80 µg of polyclonal goat anti-ricin antibodies by 6 h post-intoxication (Fig. 4B) but about 30% lethality were observed when antibodies were applied at 7 h post-intoxication. Increasing the time interval between intoxication and rescue such as adding antibodies at 8 h or later post-intoxication increased the mortality rates.

## Discussion

Ricin, one of the most potent biological toxins and U.S. government listed Select Agents, is a serious bioterrorism concern. The toxin can potentially be delivered via inhalation (in), oral or systemic routes of intoxication. Data for human intoxication have been scarce [3], [5]. Using the mouse as a model for intoxication, we determined the LD_50_ of pure ricin to be 29 mg/kg when administered orally, compared to 0.023 mg/kg for the LD_50_ observed when administered ip (Table 1). The oral LD_50_ was approximately 1200 times higher than ip-treated mice, consistent with previous data [3], [26], [27]. Even less ricin, 0.008 mg/kg, or about 3600 times less toxin, is required to cause lethality in mice when introduced iv. The differences in toxicity of the different routes illustrate the differences in how the toxin is absorbed and processed *in vivo*. Gastric secretions, the intestinal epithelia and mucosal immunity may present strong barriers to ricin absorption and distribution as indicated by the relatively large doses of ricin needed to cause death upon oral intoxication [10]. Ricin is much more readily absorbed when introduced via any of the other three routes of intoxication (in, ip or iv) where close proximity to the bloodstream and more accessible cells are thought to be available [3].

Despite large differences in LD_50_ values, a comparison of the histopathology of mouse organs or tissues showed that ip and orally intoxicated mice displayed similar lesions in lymphoid tissues (Fig. 1 and data not shown). Occasional intestinal epithelial or mucosal damage was observed in orally treated animals as manifestation of route specific damage. The susceptibility to ricin toxicity may also vary depending on the animal host. In studies using the rat, lesions were observed in the liver or kidneys, whereas no such detectable lesions were observed in the same organs in mice [3], [28]. This may be partly due to the presence of different cell receptors of different animal species capable of binding ricin [29].

We sought to compare toxicity of pure and crudely prepared ricin extracts, as it is likely that crudely prepared ricin could be used in either intentional or unintentional intoxications. Crude ricin extracts contain many other proteins such as the less toxic agglutinin or RCA-I and other superantigens [30]. However, under our experimental conditions, oral and ip toxicities of pure and crude ricin were similar when adjusted for ricin content and no discernible differences in histopathology were observed (unpublished data). Presumably, the lethality observed and visible signs of lesion are caused mainly by ricin.

Because of the high potency of ricin, very little toxin is required to cause illness. The small amount present in animal biological samples, such as sera and bodily wastes, makes diagnosis of intoxication difficult. We tested a new IPCR assay for use as a new diagnostic tool for ricin. Mouse intoxication models were used in an effort to simulate the detection of ricin in human biological samples. Sera and feces from mice treated with known quantities of ricin were processed and the concentration of ricin in samples was determined using both IPCR and traditional ELISA assays. Table 3 showed that the detection threshold for ricin in serum and feces was 1 pg/mL by IPCR, and 10 ng/mL by ELISA; the IPCR assay was therefore 10,000-fold more sensitive than its comparable ELISA assay. The concentration of ricin in feces varied from animal to animal but the general trend showed initial appearance of ricin at about 2 hr post inoculation (hpi), peaking at approximately 6 hpi (Table 4). After 24 hpi, we could not detect the ricin in feces in mice dosed with 200 µg/mouse or less. Mice dosed with 400 µg/mouse or more were mildly positive for presence of ricin in feces at 24 hpi. No ricin was detected in the feces of mice dosed intravenously. Thus, it is possible to detect ricin in feces samples from mice treated with sub-lethal amounts of ricin hours to a day after ingestion.

The high sensitivity of the IPCR assay allowed the evaluation of the toxicokinetic behavior of ricin following systemic intoxication in mice. The toxicokinetics of ricin in mouse sera was shown to be biphasic (Fig 3). The early phase or alpha half-life (t_1/2_α) was determined to be 4 min (Fig 3A). This phase of rapid toxin decline likely represents the quick distribution and absorption of toxin by tissues. A slower decline phase or beta half-life (t_1/2_β) follows the t_1/2_α; this phase was determined to be about 83 min (Fig 3B). The t_1/2_β likely represents clearance of excess toxin from the blood, likely when tissues have absorbed as much ricin as possible; excess toxin is cleared slowly from the blood stream by metabolism or via unknown mechanisms [3], [31]. Following extrapolation of the sera data, about 2.3 µg of ricin was detected in sera at time 0 (Fig. 3A). This constitutes about 50% of the injected ricin and is consistent with recovery amounts detected by others. The rest of ricin is thought to be associated with cells and thus not detectable in the sera [12], [13]. Previous attempts to determine ricin toxicokinetics in mice using ELISA methods only showed a linear degradation over 4 h [12], [13]. No early times (before 1 h) were measured, so the t_1/2_α was likely not observed. However, we believe the differences in detection methodology, starting concentrations of toxin and timing of samplings, could have contributed to the differences in toxicokinetic measurements. Our detailed measurement of ricin content in sera at early time points post-intoxication revealed a more complete picture of toxin toxicokinetics.

A wide range of time-to-deaths was observed in mice orally dosed with 1 mg of ricin (Fig. 4B – PBS). This suggested a wide time frame in which ricin could be absorbed by mice and thus the difficulty in accurately detecting systemic ricin following oral intoxication. The absorption rate of ricin would depend on the individual animal, toxin clearance or degradation by gastric secretions in the intestinal tract, or natural susceptibility of the animal. Toxin may also be absorbed differently depending on the amount or quality of food remaining in an animal's stomach, as extra food components such as certain sugars may inhibit toxin absorption [32].

To estimate the time period when ricin molecules travel from the intestinal tract to the blood stream, we performed an antibody neutralization assay for mice dosed orally with a lethal dose of ricin. Antibody neutralization assays have been applied previously to mice dosed with BoNT [25]. Decrease of toxin in sera over time matched the decrease in antibody neutralization potential of anti-toxin monoclonal antibodies administered at subsequently later times post-intoxication. In this study, a similar neutralization assay was performed with iv ricin-intoxicated mice. Complete protection was observed when polyclonal antibodies against ricin were administered 30 min prior to ricin administration (Fig 4A) showing clearly that when enough antibodies are in place in the mouse, complete clearance of toxin occurs. Decreased protection was observed when antibodies were applied post-ricin, even as early as 2 min post-intoxication. Incomplete rescue of mice from death correlated well with the ricin serum toxicokinetic data, which showed that ricin was sequestered from the bloodstream at a t_1/2_α of 4 min (Fig. 3). Increased delay of antibody administration after ricin introduction led to a faster time-to-deaths, suggesting that more ricin was absorbed preventing neutralization by antibodies. Thus an antibody protection assay can give a reasonable estimation of serum transit for toxins.

Orally intoxicated mice were assessed similarly using this antibody neutralization assay. Intoxicated mice were protected from death when antibodies were introduced up to 6 h post- intoxication (Fig. 4B), suggesting that it took at least 6 h to traffic significant amount of ricin from the mouse stomach to the bloodstream. Some lethality was observed when antibodies were added at 7 h post-intoxication, indicating that for some mice, enough ricin reached the bloodstream and was absorbed into tissues between 6 and 7 h post intoxication, before antibody administration. Increasing the time between ricin challenge and antibody administration decreased the percentage of survival.

Most ricin ingested from the oral route would be degraded in the intestinal tract or shed as waste. Ricin distribution in organs was previously determined by localization of radiolabelled toxins [12], [13]. Most ricin was found in the liver, spleen and the adrenal cortex of mice following iv intoxication. However, no histopathology has been observed in the liver where most ricin is found. It is likely that the liver served to quickly metabolize ricin and contributed to the quick elimination of toxin from sera.

Ricin is not detected in urine, as most large protein molecules are usually filtered by the kidneys prior to urine production. Instead, a less toxic alkaloid ricinine, present in crude castor preparations [33], is secreted with urine and can be detected in the urine or gastric secretions by mass spectrometry techniques [29]. Currently, ricinine is used as a surrogate marker for ricin intoxication and diagnostic tests for it are available for use in animal and human samples.

This study validated the use of a novel IPCR method in the detection of ricin in mouse sera and feces, providing a framework for future diagnostic use of this assay in real human biological samples. Improvements on the toxicokinetics measurements and the determination of pathological effects of ricin following systemic and oral intoxication will provide a better understanding of the etiology of intoxication. The data obtained here will help in the future design of more effective diagnostics and therapeutic methods for ricin poisoning. In addition, we believe the methods developed herein could also be adapted for use as new diagnostic and research tools for other protein toxins as well.
